# First Pediatric Pyeloplasty Using the Senhance^®^ Robotic System—A Case Report

**DOI:** 10.3390/children9030302

**Published:** 2022-02-22

**Authors:** Juergen Holzer, Peter Beyer, Florian Schilcher, Clemens Poth, Dietmar Stephan, Christian von Schnakenburg, Wim van Gemert, Ludger Staib

**Affiliations:** 1 Department of Pediatric Surgery, Klinikum, D-73730 Esslingen, Germany; j.holzer@klinikum-esslingen.de (J.H.); p.beyer@klinikum-esslingen.de (P.B.); 2 Department of General and Visceral Surgery, Klinikum, D-73730 Esslingen, Germany; f.schilcher@klinikum-esslingen.de (F.S.); c.poth@klinikum-esslingen.de (C.P.); 3 Department of General and Visceral Surgery, Marienkrankenhaus, D-57072 Siegen, Germany; dietmar.stephan@stephan-siegen.de; 4 Department of Pediatrics, Klinikum, D-73730 Esslingen, Germany; c.schnakenburg@klinikum-esslingen.de; 5 Department of Pediatric Surgery, University of Maastricht, 6202 AZ Maastricht, The Netherlands; wim.van.gemert@mumc.nl

**Keywords:** Senhance^®^, surgical robot, pyeloplasty, pediatric

## Abstract

A pediatric robotic pyeloplasty has been performed with the Senhance^®^ robotic system for the first time in January 2021 on a 1.5-year-old girl with symptomatic ureteropelvic junction stenosis. A Senhance^®^ robotic system (Asensus Surgical^®^ Inc., Durham, NC, USA) with three arms and 5 mm instruments was used, providing infrared eye tracking of the 5 mm camera and haptic feedback for the surgeon, facilitating suturing of the anastomosis and double-J stent insertion. The robotic surgery lasted 4.5 h, was uneventful and successful, without recurrence of the ureteropelvic junction obstruction after six months, and with normal development of the patient’s growth and organ function. The use of the robotic system was shown to be safe and feasible; long term follow-up will be conducted subsequently in pediatric surgery.

## 1. Introduction

Minimally-invasive procedures have been performed in pediatric surgery for the last fifteen years covering a broad portfolio in abdominal, visceral, gynecological, and urological surgery [1].

Ureteropelvic junction obstruction (UPJO), a common cause of pediatric hydronephrosis, is present in 1 in 1000 to 2000 newborns [2], sometimes with a delay of the diagnosis up to several years, and with impaired kidney function. Treatment depends on severity of obstruction and consists of decompression or resection of the pyelon and anastomosis between the ureter and pyelon with a stent (often a double-J stent) inserted [3]. The gold standard of treatment is open pyeloplasty, which is successful in >90% of the patients. In 2002, the first laparoscopic pyeloplasty was performed by the group of Kearns at Chicago University [4]. The laparoscopic technique has been adapted to the robotic approach, combining technical and outcome benefits of both techniques. Some surgeons have applied their open technique directly to robotic surgery without laparoscopic experience, due to a steep learning curve. Pyeloplasty is the most commonly performed procedure in pediatric robotic surgery [5].

The majority of the current published outcome data of the robotic-assisted pediatric pyeloplasty is with reference to the Da Vinci robotic platform (Intuitive Surgical Inc., Sunnyvale, CA, USA). However, a second robotic system, the Senhance^®^ platform, was introduced in 2016 with Conformité Européenne (CE) and Food and Drug Administration (FDA) approval, manufactured by Asensus Surgical Inc. (Naderhan, NC, USA), former Transenterix Inc. (Naderhan, NC, USA). For the Senhance^®^ robotic system, safety and feasibility have been shown for various procedures performed in adults in general and visceral surgery, gynecology, and urology [6,7,8,9]. In general pediatric surgery, the first Senhance^®^ procedures were performed at Maastricht University/Netherlands, in November 2020 by the group of W. van Gemert. In their post-market surveillance study, they evaluated the usability, safety, and efficacy of the Senhance^®^ robotic system in children at the age of one year or older or with a body weight of 10 kg and above. However, wet lab data showed that the Senhance^®^ can well be used in small cavities, for instance, in operations on small children using 3 mm robotic instruments [10,11].

To our knowledge, the pediatric pyeloplasty performed in our hospital was the first one worldwide using a Senhance^®^ robot in a child and performing an anastomosis between the pyelon and the ureter.

## 2. Patient and Method

### 2.1. System

The robotic system Senhance^®^ (Asensus Surgical Inc.) with three arms had been installed in Klinikum Esslingen, Germany in January 2020. The Senhance^®^ surgical robot consists of three (to four) separately movable robotic arms and a separate console where the surgeon is sitting non-sterile and telemanipulating the system via handles with sensors, similar to those used in laparoscopic surgery. The system allows the surgeon to navigate the camera via the eye-tracker. The Senhance^®^ works in a fulcrum mode, i.e., right movement of the handle leads to left movement within the abdominal cavity. However, it allows movement in “opposite fulcrum mode” if the surgeon changes compensation angles. Thus, the system is versatile and flexible, and the robot arms can be used on either side of the patient. Monopolar and bipolar 5 mm instruments can be attached to the robot arms via a magnetic adapter that allows rapid detachment in case of emergency. The robot arms can operate within three velocity modes (high/medium/low) to support either very subtle or faster tissue preparation. The system is only operating if the surgeon‘s left foot presses the foot pedal (clutch), and if each three fingers of both hands are in contact with both sensors of the handles. The robots warns the surgeon in time, if the pressure or the traction of the instruments on the tissue are too high (“warning exceeding force”), or if the robot arms tend to reach their motion limits (“warning limited motion”). The system had been developed from a former early robot model called TELELAP ALF-X and which had been described for robotic inguinal hernia repair [12].

Surgeons and nurses had been trained previously in a structured team training program in Milan, Italy, provided by Asensus Surgical Inc. The training included two days of dry lab training and one day of wet lab training, including a written test for the surgeons before obtaining the certificate mandatory before operating the robot. The novel robotic surgery program was implemented according to a three-step protocol (simple/medium/advanced procedures) in the departments of visceral and thoracic surgery, one year later in pediatric surgery. During the first procedures at each step, a senior proctor surgeon (D.S.) and a technical specialist were present.

### 2.2. Patient

**Figure 1:**
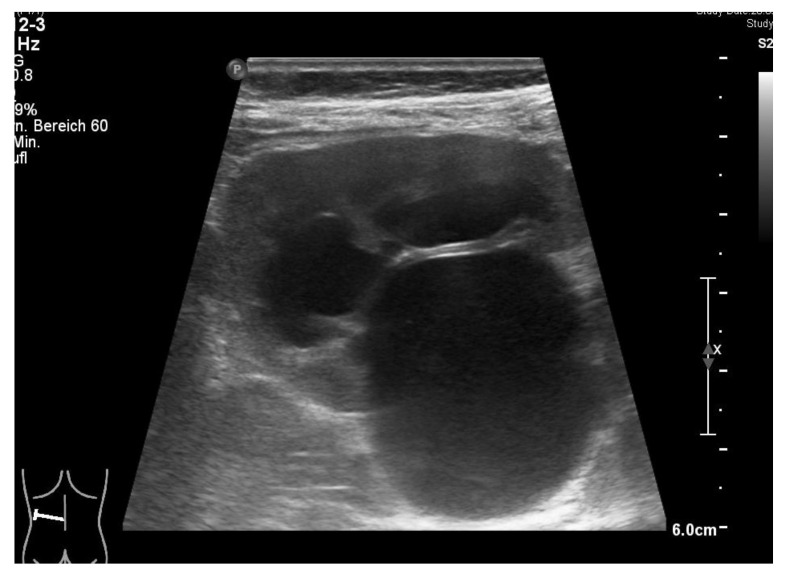
Ultrasound test of 1.5-year-old girl (19 May 2020) presenting with recurrent febrile urinary tract infections shows dilatation of the pyelon and hydronephrosis of the left kidney (courtesy of Dr. A. Longin, Klinikum Esslingen).

We present the case of a girl with a first proven febrile urinary tract infection at the age of 15 months. Her history revealed several previous episodes of fever of unknown origin, some treated with antibiotics, but no additional comorbidity. On assessment, she had a raised c-reactive protein (CRP, maximum 133 mg/L), a normal serum creatinine (0.37 mg/dL), and an isolated left-sided third grade hydronephrosis, suggestive of ureteropelvic junction stenosis with a 2.8 cm pelvic dilatation (Figure 1). Catheter urine showed >1000 leucocytes per high power field, and *Escherichia coli* was isolated (10^5^/mL). She responded well to parenteral cefuroxime and was discharged after five days with oral prophylactic trimethoprim.

**Figure 2:**
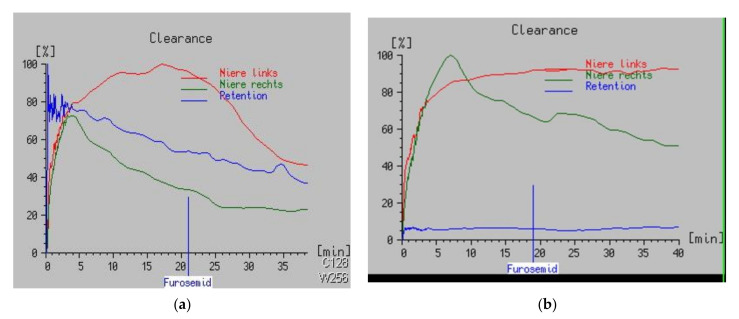
(**a**) In the kidney scintigraphy test, a retention kidney on the left side with borderline reaction on furosemide stimulation and excretion half-time of 17:39 min implicated a ureteropelvic junction obstruction (UPJO) (18 June 2020). Norm values: normal < 10 min, suspicious 10–20 min, abnormal > 20 min (courtesy of Dr. P. Zimmer, Klinikum Esslingen). (**b**) Repeated kidney scintigraphy test, constant retention kidney on the left side. Minor reduction of urinary clearence (left side 40%, right side 60%) (5 November 2020). Indication for surgery (courtesy of Dr. P. Zimmer, Klinikum Esslingen).

At the age of 17 months, a renal isotope scan with Tc-99m-mercaptoacetyltriglycine (MAG3) showed similar bilateral function (right 54% versus left 46%) and a left-sided renal accumulation curve, yet with good response to diuretic challenge (50% reduction in 17.5 min) (Figure 2a). On ultrasound, pelvic dilatation had not increased, and voiding cystourethrography showed no reflux.

After an uneventful clinical course and a strategy of “wait and see”, renal scintigraphy was repeated at age 18 months. Left-sided function had decreased to 40%, and excretion no longer responded to furosemide (Figure 2b). Ultrasound persistently showed a dilated left renal pelvis (2.2 cm) and renal calyces, and a narrowed parenchyma (5 mm). Thus, the elective robotic left Anderson–Hynes pyeloplasty was planned at the age of 18 months, and informed consent of the parents was obtained.

The robotic set-up and the steps of the procedure were discussed in detail with experienced colleagues (W.v.G., D.S.) and the clinical specialists of Asensus Surgical. A 5 mm 4 K camera was kindly provided by Karl Storz (Tuttlingen, Germany). On 20 January 2021, robotic pyeloplasty was performed (J.H., P.B., F.S., C.P., L.S.). The procedure was proctored by a robotic-experienced surgeon (D.S.).

### 2.3. Procedure

We used the Senhance^®^ robotic system with three arms and a 30-degrees 5 mm 4 K video camera system (Karl Storz, Tuttlingen, Germany), connected with a newly designed 5 mm adapter to the Senhance^®^. The patient was operated on under general anesthesia in a supine position and tilted 20° to the right side. Single-shot antibiotic prophylaxis was given with trimethoprim 25 mg.

The first 5 mm incision was made at the umbilicus for the 5 mm camera, and CO_2_ was insufflated at a maximum pressure of 12 mm Hg. The laparoscopy revealed a regular situs without adhesions.

**Figure 3:**
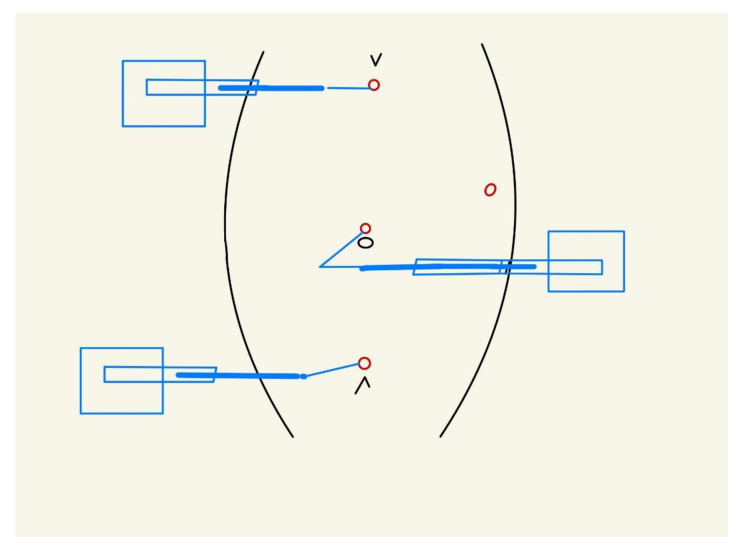
Placement of ports and Senhance^®^ robot arms in a three-arm setting. The right robotic working arm is placed at the patient’s right side with a suprapubic 5 mm port; the left robotic working arm is placed at patient’s right side with a subxiphoid 5 mm port. The camera arm is placed at the patient’s left side with a 5 mm umbilical port, and works “overhead” with the instrument coming from the patient’s right side. One additional auxiliary 5 mm port is placed at the left abdominal wall.

**Figure 4:**
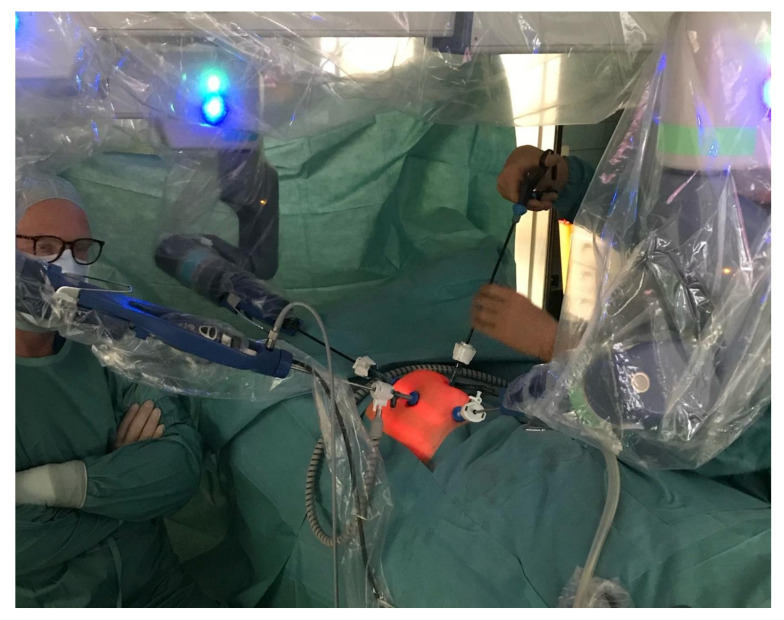
Robotic left sided Anderson–Hynes pyeloplasty (21 January 2021). Robotic arms have been docked (blue docking adapters) to the camera and two instruments; one auxiliary port is placed in the left hypogastric abdomen. Note the camera arm working “overhead” from the patient’s right side with the robot arm base being placed on the patient’s left side (see Figure 3).

The left-arm 5 mm trocar was placed at the epigastrium, the right-arm trocar suprapubically (Figure 3 and Figure 4). One arm was placed on the patient’s right side and two arms on the patient’s left side.

**Figure 5:**
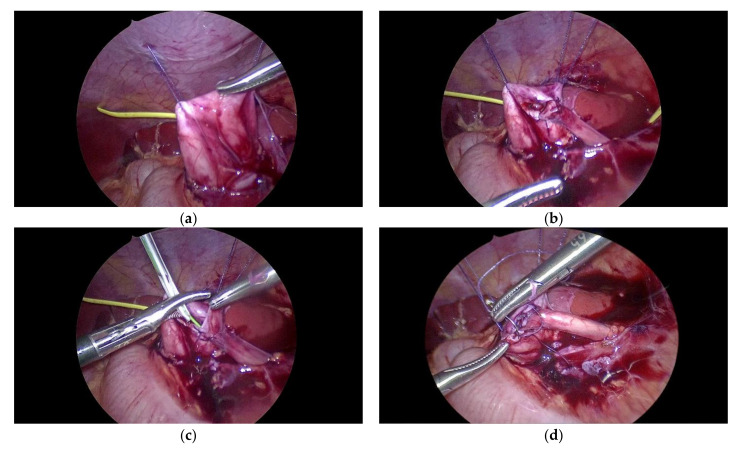
(**a**) Exposition of the ureteropelvic junction obstruction with stay sutures. (**b**) The posterior wall of the anastomosis between the pyelon and left ureter is finished. (**c**) Insertion of the pigtail catheter after the posterior wall of the anastomosis had been completed. (**d**) The anterior wall of the anastomosis is finished with eight single stiches of Vicryl 5-0.

The camera arm was positioned in the supraumbilical port, the left-hand arm in the epigastric port, and the right-hand arm was positioned suprapubically. For the 5 mm 4 K camera, we had received a newly remodelled adapter which is stronger than the former model and gives a very stable picture. The left colon was mobilized and the left kidney was exposed. We added a fourth auxiliary 5 mm port on the left flank to lift up the kidney, to expose the pyelon and to insert the sutures later on. The pyelon was dissected from the left ureter below the vessels and transposed in front on the vessels for the anastomosis between the pyelon and the spatulated ureter (Figure 5a,b). A double-J stent (4.2 French, 15 cm of length) was inserted transcutaneously (Figure 5c). The ventral circumference of the anastomosis was completed with eight single stiches of Vicryl 5-0 (Figure 5d). A drain was placed next to the anastomosis. The operation lasted 4.5 h and was uneventful.

## 3. Results

**Figure 6:**
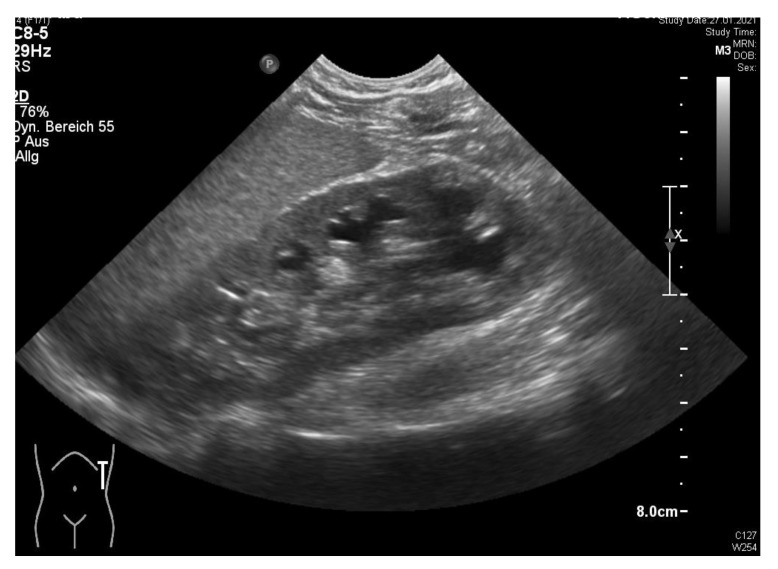
Ultrasound test after left-sided robotic pyeloplasty before dismission shows normal kidney without dilatation of the pyelon (courtesy of Dr. A. Longin, Klinikum Esslingen).

Postoperatively, the patient was transferred to the regular pediatric ward. On POD-2, she had one event of intermittent fever (38.9 °C); antibiotic therapy was switched from cefuroxim to cefotaxime, according to the antibiogram with *E. coli* in the urine test. On POD-4, the urinary catheter was removed, after the ultrasound control (Figure 6) had revealed a regression of the pyelon dilatation and a ureteral catheter correctly placed. On POD-6, the abdominal drain was removed. On POD-7, the patient was dismissed with normal kidney function and a normal CRP (2.7 mg/L). Histology revealed a chronic fibrosis of the resected left pyelon and of the left ureter.

Antibiotic therapy with oral cefpodoxime 2 × 60 mg per day was continued for 10 days, and subsequently reduced to 20 mg per day until removal of the ureter stent after six weeks. After follow-up for six months, there were no signs of recurrent ureteropelvic junction obstruction. The patient showed normal developmental growth and organ function.

Follow-up: Six months later, no clinical or sonographic signs of recurrent ureteropelvic stenosis were present. A control renal isotope scan with Tc-99m-mercaptoacetyltriglycine (MAG3) showed a consistently regular bilateral function (right 44% versus left 56%; regular range 45–55%) without left-sided renal accumulation. Clinical and sonographic monitoring will be carried out every six months.

## 4. Discussion

To our knowledge, this is the first reported robotic pyeloplasty in a child using a Senhance^®^ robotic system. Earlier reports of pediatric robotic pyeloplasty had been based on the Da Vinci robotic platform that contains one cart with four robotic arms attached [1] instead of single moveable arms.

A setting with four ports was performed in our approach, using three robotic ports and one auxiliary port. An alternative could have been the use of four robotic arms, but still the needles and sutures would have needed to be placed into the abdomen. We find the auxiliary 5 mm port in the left upper abdomen helpful for inserting and extracting sutures, exposing the kidney in addition to stay sutures, and for lavage and suction or cutting sutures.

The camera arm in the supraumbilical port was well placed, while the left-hand arm in the epigastric port was ca. 2 cm too far cranially. The right-hand arm which had been positioned suprapubically was also ca. 2 cm too far caudally, because the arm was too close to the right leg. Arm collisions were not observed, although arm collisions can be a problem during robotic surgery, as they are in other robotic systems. Using a 5 mm 4 K camera was sufficient to perform safe single-stiches for the anastomosis with a 5-0 suture, although we usually use a 10 mm 3D camera system (Olympus Inc., Tokyo, Japan) in adults to obtain a perfect visualization of the operation field. We found the camera movement with the infrared eyetracker of the Senhance^®^ very comfortable and stable to use. The haptic feedback was reminiscent of a rubber band traction and facilitated performing fine sutures in the exact position and with the appropriate tension of the knots.

We had chosen 5 mm instruments, because we are more familiar with them. However, there are 3 mm instruments available now. Perhaps these would work as well or even better in the setting described [13]. Articulating 5 mm instruments that are currently being introduced into Senhance^®^ robotic surgery [14] would most likely facilitate the fine preparation and suturing steps even better. Up to now, robotic surgery had been limited to 8 mm instruments which seems to be inadequate for small children, because of larger abdominal incisions and higher abdominal trauma, with the potential risk of larger scars, hernias or adhesions. Therefore, the novel robotic technology had rarely been offered to small children [15,16,17]. The development of a new robotic system (Senhance^®^ by Asensus Surgical, Inc.) for robot-assisted or computer-assisted laparoscopy which involves 3 mm instruments and articulating 5 mm instruments will enable surgeons to perform robotic procedures in small children, not only with classical abdominal and thoracic surgery procedures, but also in pediatric gynecology, pediatric neurosurgery, and pediatric ETN surgery. Perhaps single-port procedures will become possible using three small-diameter instruments in one port instead of three ports with separate incisions. The group of Bergholz has evaluated this approach in a preclinical study [10] (Bergholz, 2020): in their avital model (“box trainer”), they demonstrated that robotic procedures in small cavities with a minimal volume of minimal 92 mL were feasible. Before introduction of the Senhance^®^ pediatric robotic surgery into daily routine, further studies in small children will be necessary to prove safety and efficacy in different pediatric specialties.

As an additional advantage of the Senhance^®^ robotic system compared to ordinary minimal-invasive surgery, besides the eyetracker, we see the relaxed sitting position of the surgeon with high precision, tremor-free movement of the instruments and the camera, which would most likely increase preparation precision and minimize the surgical trauma. Correspondingly, it has been reported that the length of stay in the hospital was 14% shorter in children below five years when surgery was performed using a robotic system. Length of stay had been 2.0 days after open vs. 2.4 days after laparoscopic vs. 1.8 days after robotic surgery, while similar complication rates of 2.1 vs. 2.2 vs. 3% had been reported [18]. In contrast, our length of stay was seven days regardless of the patient’s uneventful course, because length of stay in German/European hospitals differs in general significantly from U.S.-reported data, due to differences in the medico-economic systems. For the Da Vinci system, *n* = 13 pediatric pyeloplasties with a mean hospital length of stay of 5.85 days and a mean surgery time of 111.54 min has been reported in a German university hospital [19]. This compares well to the data reported with the Senhance^®^ robotic system, although operating times were much shorter in this reported series, most likely due to the learning curve of the surgeons. The reported complication rate was 15.38%, and a recurrence rate of 7.69% was observed. The lack of specific instruments for pediatric surgery and the size of the instruments were mentioned as potential disadvantages, while the procedure was feasible and safe, and parents seemed to be satisfied. Costs of the procedure were not mentioned [19]. Costs of robot-assisted surgical procedures are in general higher than in regular laparoscopic surgery, due to investment and maintenance costs of the robot. In a critical literature review of comparative outcomes reported for the least invasive management of ureteropelvic obstruction in children, a Spanish group found two major drawbacks of robotic-assisted laparoscopic pyeloplasty in children: costs and the size of instruments [20]. This might be true for the Da Vinci robotic system. For the Senhance^®^ system however, 3 and 5 mm instruments can be used, as mentioned before. In addition, costs seem to be lower: for the Senhance^®^ system, we calculated a fee of 1500.00 € or less per robotic procedure, based on rental, service and procedure costs. The reimbursement by the health care provider was 8192.00 € for the pediatric pyeloplasty described (DRG code L04A, relative weight 1.956).

Further evaluation, also utilizing 3 mm surgical instruments and articulating 5 mm instruments as well, will be necessary to define the additional benefit of the Senhance^®^ robotic platform used in pediatric ureteropelvic junction obstruction and further procedures.

## Data Availability

Data was obtained from the clinical information system of Klinikum Esslingen/Germany, including patient’s clinical charts and image recording system.

## References

[1] Morales-López R.A., Pérez-Marchán M., Pérez Brayfield M. (2019). Current Concepts in Pediatric Robotic Assisted Pyeloplasty. Front. Pediatr..

[2] Vemulakonda V.M., Wilcox D.T., Crombleholme T.M., Bronsert M., Kempe A. (2015). Factors associated with age at pyeloplasty in children with ureteropelvic junction obstruction. Pediatr. Surg. Int..

[3] Pogorelić Z., Brković T., Budimir D., Todorić J., Košuljandić D., Jerončić A., Biočić M., Saraga M. (2017). Endoscopic placement of double-J ureteric stents in children as a treatment for primary hydronephrosis. Can. J. Urol..

[4] Gundeti M.S., Kearns J. (2014). Pediatric robotic urologic surgery-2014. J. Indian Assoc. Pediatr. Surg..

[5] Howe A., Kozel Z., Palmer L. (2017). Robotic surgery in pediatric urology. Asian J. Urol..

[6] Kastelan Z., Hudolin T., Kulis T., Knezevic N., Penezic L., Maric M., Zekulic T. (2021). Upper urinary tract surgery and radical prostatectomy with Senhance^®^ robotic system: Single center experience—First 100 cases. Int. J. Med. Robot. Comput. Assist. Surg..

[7] Samalavicius N.E., Janusonis V., Siaulys R., Jasėnas M., Deduchovas O., Venckus R., Ezerskiene V., Paskeviciute R., Klimaviciute G. (2020). Robotic surgery using Senhance® robotic platform: Single center experience with first 100 cases. J. Robot. Surg..

[8] Rumolo V., Rosati A., Tropea A., Biondi A., Scambia G. (2019). Senhance robotic platform for gynecologic surgery: A review of literature. Updates Surg..

[9] Stephan D., Sälzer H., Willeke F. (2018). First Experiences with the New Senhance Telerobotic System in Visceral Surgery. Visc. Med..

[10] Bergholz R., Botden S., Verweij J., Tytgat S., Van Gemert W., Boettcher M., Ehlert H., Reinshagen K., Gidaro S. (2020). Evaluation of a new robotic-assisted laparoscopic surgical system for procedures in small cavities. J. Robot. Surg..

[11] Krebs T.F., Egberts J.-H., Lorenzen U., Krause M.F., Reischig K., Meiksans R., Baastrup J., Meinzer A., Alkatout I., Cohrs G. (2021). Robotic infant surgery with 3 mm instruments: A study in piglets of less than 10 kg body weight. J. Robot. Surg..

[12] Schmitz R., Willeke F., Darwich I., Barr J., Scheidt M., Saelzer H., Darwich I., Zani S., Stephan D. (2019). Robotic inguinal hernia repait (TAPP)—First experience with the new Senhance robotic system. Surg. Technol. Int..

[13] Montlouis-Calixte J., Ripamonti B., Barabino G., Corsini T., Chauleur C. (2019). Senhance 3-mm robot-assisted surgery: Experience on first 14 patients in France. J. Robot. Surg..

[14] Stephan D., Darwich I., Willeke F. (2020). First Clinical Use of 5 mm Articulating Instruments with the Senhance Robotic System. Surg. Technol. Int..

[15] Baek M., Silay M.S., Au J.K., Huang G.O., Elizondo R.A., Puttmann K.T., Janzen N.K., Seth A., Roth D.R., Koh C.J. (2018). Does the use of 5 mm instruments affect the outcomes of robot-assisted laparoscopic pyeloplasty in smaller working spaces? A comparative analysis of infants and older children. J. Pediatr. Urol..

[16] Ballouhey Q., Clermidi P., Cros J., Grosos C., Rosa-Arsène C., Bahans C., Caire F., Longis B., Compagnon R., Fourcade L. (2018). Comparison of 8 and 5 mm robotic instruments in small cavities: 5 or 8 mm robotic instruments for small cavities?. Surg. Endosc..

[17] Finkelstein J., Levy A., Silva M., Murray L., Delaney C., Casale P. (2015). How to decide which infant can have robotic surgery? Just do the math. J. Pediatr. Urol..

[18] Chan Y.Y., Durbin-Johnson B., Sturm R.M., Kurzrock E.A. (2017). Outcomes after pediatric open, laparoscopic, and robotic pyeloplasty at academic institutions. J. Pediatr. Urol..

[19] Ammer E., Kahl F. (2020). Robot-assisted (RA-)pedatric surgery: Pyeloplasty with the Da Vinci robotic system. Monatsschr Kinderheilkd.

[20] Castagnetti M., Iafrate M., Esposito C., Subramaniam R. (2020). Searchung for the least invasive management of pelvi-ureteric junction obstruction in children: A critical literature review of comparative outcomes. Front. Pediatr..

